# Ischemia reperfusion-induced metastasis is resistant to PPARγ agonist pioglitazone in a murine model of colon cancer

**DOI:** 10.1038/s41598-020-75210-6

**Published:** 2020-10-29

**Authors:** Yoshihiko Tashiro, Hiroto Nishino, Takashi Higuchi, Norihiko Sugisawa, Yasunari Fukuda, Jun Yamamoto, Sachiko Inubushi, Takeshi Aoki, Masahiko Murakami, Shree Ram Singh, Michael Bouvet, Robert M. Hoffman

**Affiliations:** 1 AntiCancer Inc, 7917 Ostrow St, San Diego, CA 92111 USA; 2 Department of Surgery, University of California, San Diego, CA USA; 3 Department of General and Gastroenterological Surgery, Showa University School of Medicine, 1-5-8 Hatanodai, Shinagawa-Ku, Tokyo, 142-8666 Japan; 4 Basic Research Laboratory, National Cancer Institute, Frederick, MD 21702 USA

**Keywords:** Cancer, Cell biology, Drug discovery, Oncology

## Abstract

Ischemia reperfusion injury (IRI) during liver-metastasis resection for treatment of colon cancer may increase the risk of further metastasis. Peroxisome proliferator-activated receptor-γ (PPARγ) activation has been observed to exert a protective effect against IRI and IRI-induced metastasis of hepatocellular carcinoma. The present study aimed to investigate the effect of the PPARγ agonist pioglitazone on tumor metastasis and liver injury following IRI in a mouse model of colon cancer. Pioglitazone (30 mg/kg weight) was administered orally 1.5 h before and 2 h after the initiation of ischemia and was orally administrated daily to mice from day 0–21. SL4-cancer cells expressing red fluorescent protein (SL4-RFP) (1 × 10^6^) were injected into the spleen. Fifteen minutes after injection, the hepatoduodenal ligament was clamped with a vessel clip, and released 5 min later. Liver, blood and tumor samples were taken from mice in order to determine if inflammation was induced by IRI. The effect of pioglitazone on liver metastasis was assessed. Furthermore, the effect of pioglitazone to control the inflammatory response during IRI progression was examined. Liver metastasis along with MMP-9 activation and the production of inflammatory cytokines were resistant to pioglitazone. Our results indicate that liver metastasis and associated inflammation in mice were resistant to pioglitazone.

## Introduction

Liver resection is the most curative treatment for malignant tumors of the liver^1^. Intraoperative intermittent hepatic pedicle clamping, the Pringle maneuver, is widely used to minimize blood loss and transfusion during liver transection. However, it has been reported that use of the Pringle maneuver resulted in ischemia/reperfusion injury (IRI) to the remnant liver by the activation of inflammatory signaling pathways and production of reactive oxygen species (ROS)^2^. Various studies suggest that IRI triggers additional metastasis development including intrahepatic and lung metastasis by multiple mechanisms (3–5). Diminishing the effect of IRI, can reduce metastasis of colorectal cancer to the liver^3,4^. However, the clinical impact of hepatic IRI on tumor recurrence remains controversial (6–17).

IRI during liver resection promotes liver micro-metastasis by liver functional failure, inflammatory cytokines and MMP activation (3–6). Peroxisome proliferator-activated receptor-γ (PPAR-γ) is a ligand-activated transcription factor belonging to the nuclear hormone-receptor superfamily. PPAR-γ has been reported to reduce IRI injury due to MMP activation^5^. Therefore, we tested whether pioglitazone, a PPAR-γ agonist could inhibit liver metastasis of colon cancer after IRI in an experimented spleen-injection metastasis mouse model.

## Materials and methods

### Animal

C57BL/6 mice (AntiCancer Inc San Diego CA) 8–10 weeks were used in this study. Mice were housed in a barrier facility on a high efficacy particulate arrestance (HEPA)-filtered rack under standard conditions of 12-h light/dark cycles. Animal studies were performed with an AntiCancer Institutional Animal Care and Use Committee (IACUC)-protocol specially approved for this study and in accordance with the principles and procedures outlined in the National Institutes of Health Guide for the Care and Use of Animals under Assurance Number A3873-1.

### Cell lines

The mouse colon cancer cell line SL4 labeled with red fluorescent protein (RFP) was established as described previously^6–9^. The SL4-RFP cell line was cultured in RPMI-1640 with 10% fetal bovine serum and 1% penicillin–streptomycin mixture. Cells were cultured 37℃ in a humidified atmosphere containing 5% CO_2_.

### Study design

**Figure 1:**
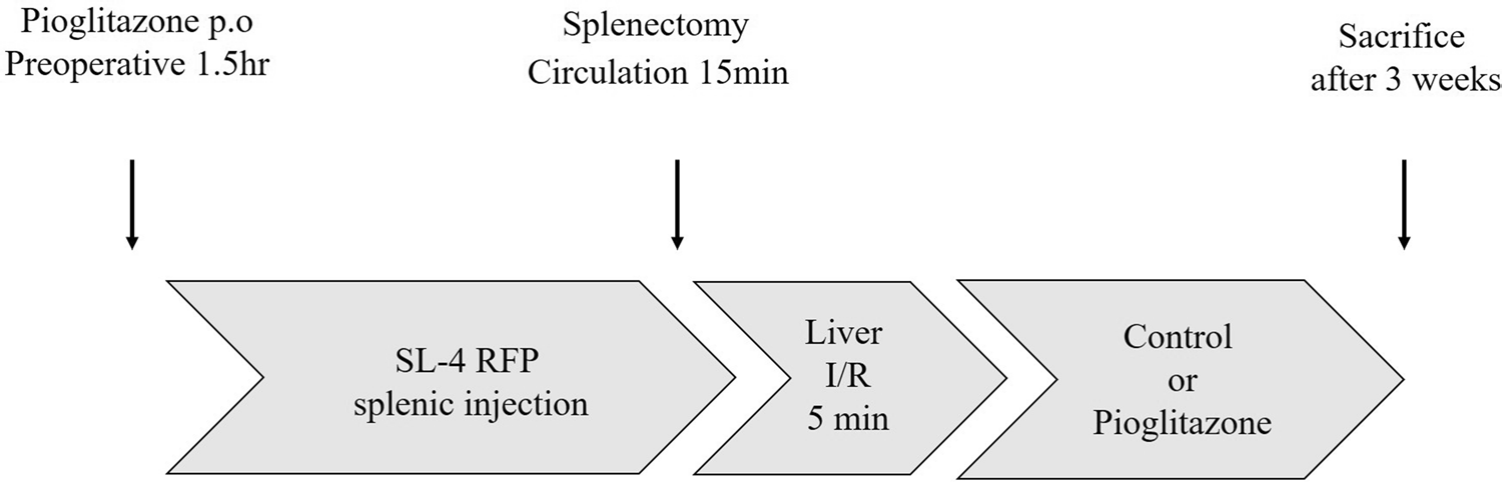
Treatment schema. Treatment protocol. Pioglitazone (30 mg/kg weight) was administered orally 1.5 h before and 2 h after the initiation of ischemia. SL4-RFP cells 1 × 10^6^ was injected into the spleen. After 15 min, the hepatoduodenal ligament was clamped with a vessel clip, which was released after 5 min. After the procedure, Pioglitazone was orally administrated daily to mice from day 0–21.

The PPARγ agonist, pioglitazone solubilized in DMSO, was orally administrated (30 mg/kg weight) daily to mice from day 0–20. Pioglitazone was administered orally 1.5 h before and 2 h after the initiation of ischemia. The IRI mouse model was modified from the method previously described^10^. Briefly, an incision was made in the skin and the left subphrenic to expose the spleen. SL4-RFP cells (1 × 10^6^) prepared in PBS (100 ul) were injected into the spleen using a 29-gauge needle. A laparotomy was then performed in the upper abdomen. Fifteen minutes after injection, the hepatoduodenal ligament was clamped with a vessel clip and released in 5 min. After releasing the clip, splenectomy was quickly performed to prevent tumor growth in the spleen. The wound was closed by 6–0 nylon thread. Mice were separated into two groups of 7 mice each: control with I/R (n = 7); pioglitazone with I/R (p.o., 30 mg/kg, 21 consecutive days, n = 7) (Fig. 1). Blood was collected by retro-orbital bleeding with the use of heparinized capillaries. White blood cells (WBCs) were counted. Serum samples were stored at -30 °C. Mice were sacrificed 21 days after I/R. Data are presented as mean ± SD.

### Peroxisome proliferator-activated receptor-γ antagonist

Peroxisome proliferator-activated receptor-γ antagonist, pioglitazone, was purchased from Cayman Chemical Company, Inc. (Ann Arbor, MI, USA).

### Histological assessment

Whole liver samples were fixed in 10% phosphate-buffered formalin for ≥ 5 days. Sections were stained with haematoxylin and eosin (H&E).

### Transaminase measurement

Serum aspartate aminotransferase (AST) and alanine aminotransferase (ALT) were measured using a transaminase CII-test Wako kit (Wako Pure Chemical Industries, Tokyo)^11^.

### ELISA

The level of cytokines was determined in serum. Samples were measured using commercially available mouse-specific ELISA kits for murine MMP-9, TNF-α, IL-6 (R&D Systems Inc., Minneapolis, MN).

### Statistical analysis

All data are presented as mean ± standard deviation (SD). Student’s t-tests were performed. Survival curves were plotted using Kaplan–Meier estimates with log rank. P < 0.05 was considered significant.

## Results

### Pioglitazone treatment after IRI

**Figure 2:**
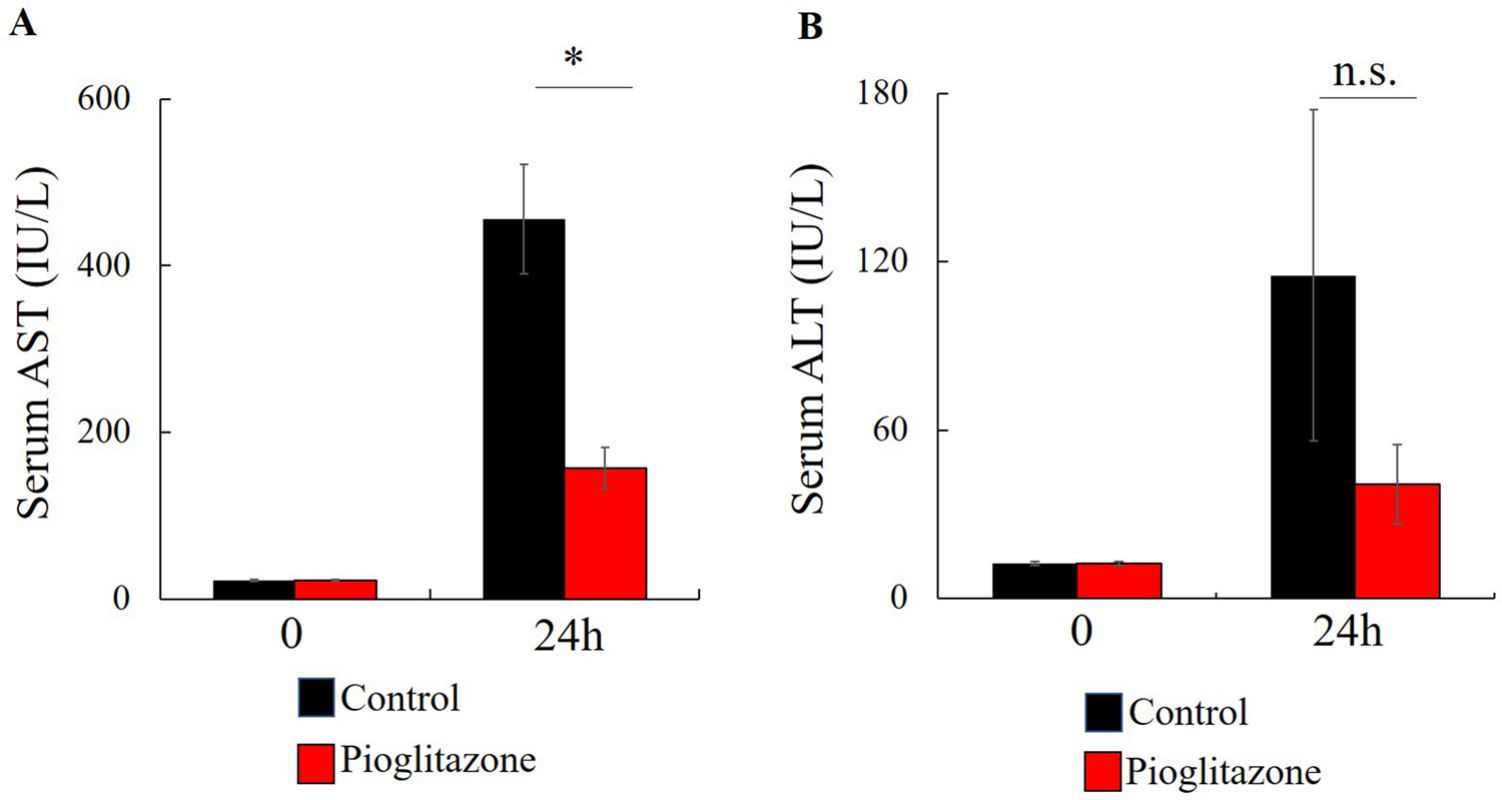
Effect of pioglitazone on liver damage after IRI. (**A**, **B**) Serum level of AST and ALT were measured with standard kits (AST and ALT; *n* = 7). Values represent the mean ± SD. *P < 0.05.

Alanine transaminase (ALT) and aspartate transaminase (AST) levels were measured to assess the extent of hepatic damage to the liver 24 h after IRI. Serum AST levels decreased after pioglitazone treatment in IRI-induced mice (Fig. 2A). Serum ALT was not affected by pioglitazone in IRI mice (Fig. 2B).

### Pioglitazone did not inhibit MMP-9 activation and inflammatory response

**Figure 3:**
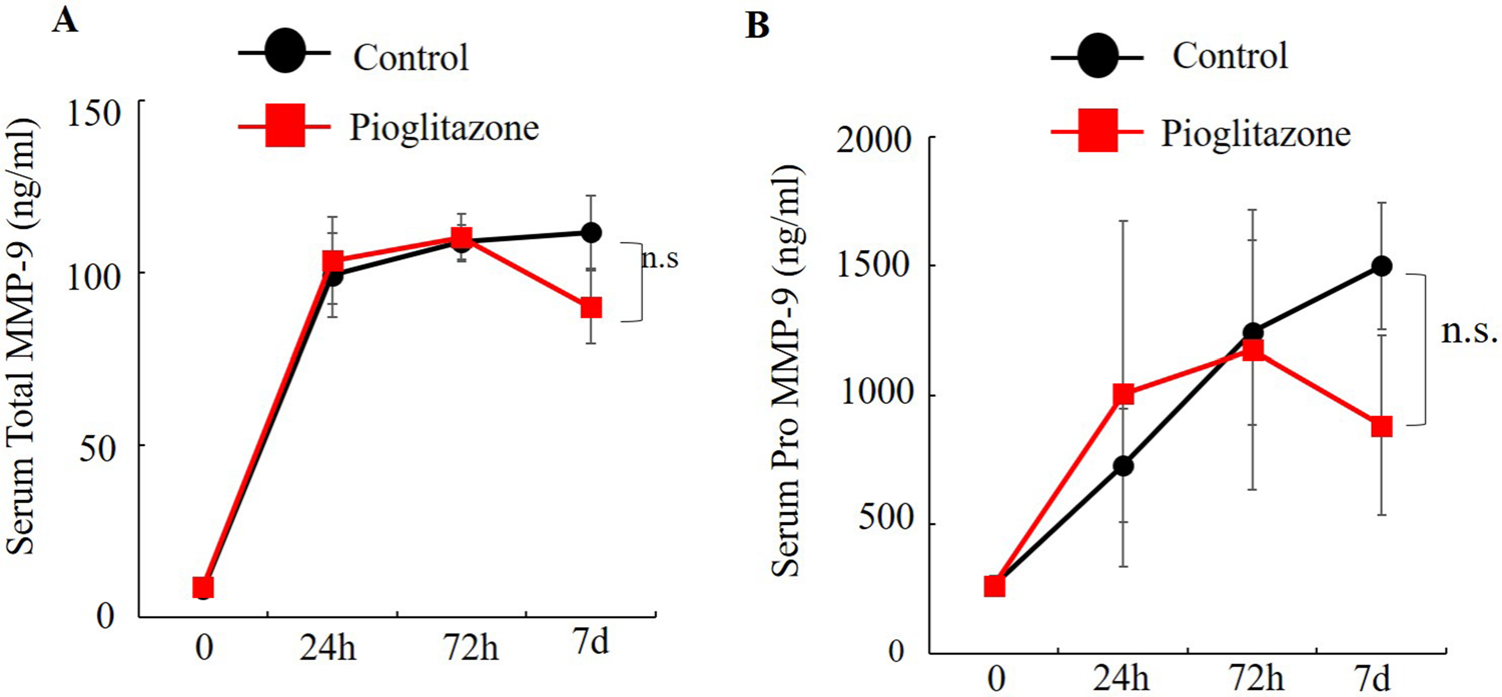
Effect of pioglitazone on MMP-9 after IRI. MMP-9 serum levels were determined by ELISA in mice treated with pioglitazone, or with vehicle (n = 5). (**A** and **B**) Values represent the mean ± SD.

**Figure 4:**
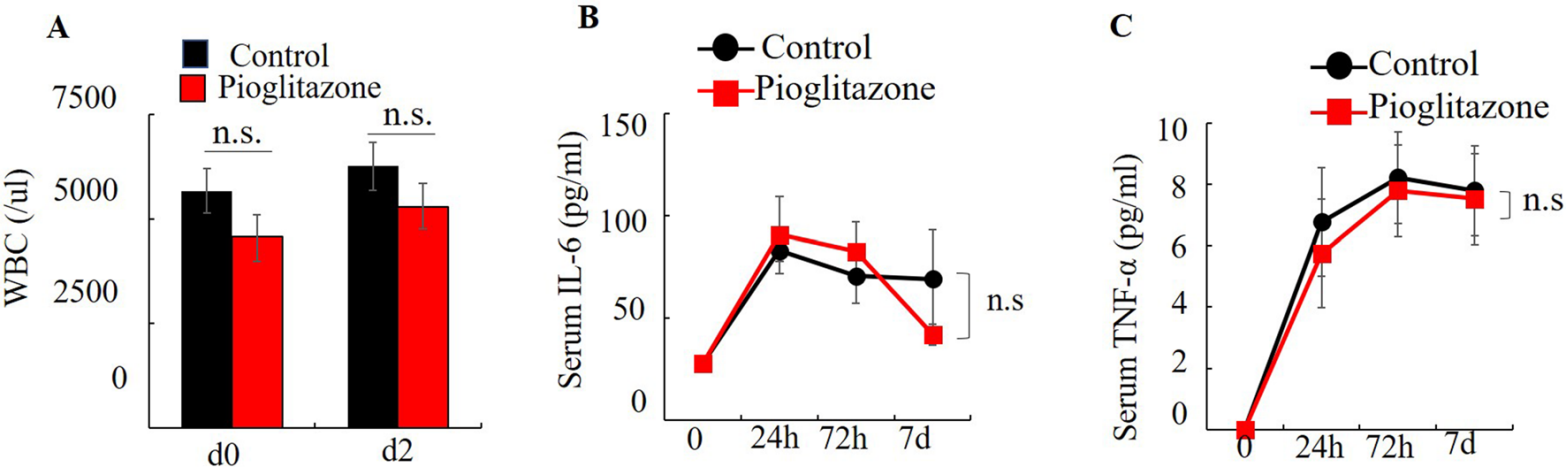
Effect of pioglitazone on inflammatory responses after IRI. The total number of white blood cells (WBC) was determined in the peripheral blood of Pioglitazone-treated or vehicle-treated mice (n = 4) (**A**). Pioglitazone did not inhibit inflammatory cytokines after IRI (n = 5) (**B** and **C**). Values represent the mean ± SD.

Pioglitazone did not inhibit MMP-9 serum levels (Fig. 3A and B). Pioglitazone also did not regulate the inflammatory response indicated by IL-6 and TNF-α levels (Fig. 4A-C).

### Pioglitazone did not inhibit liver metastasis

**Figure 5:**
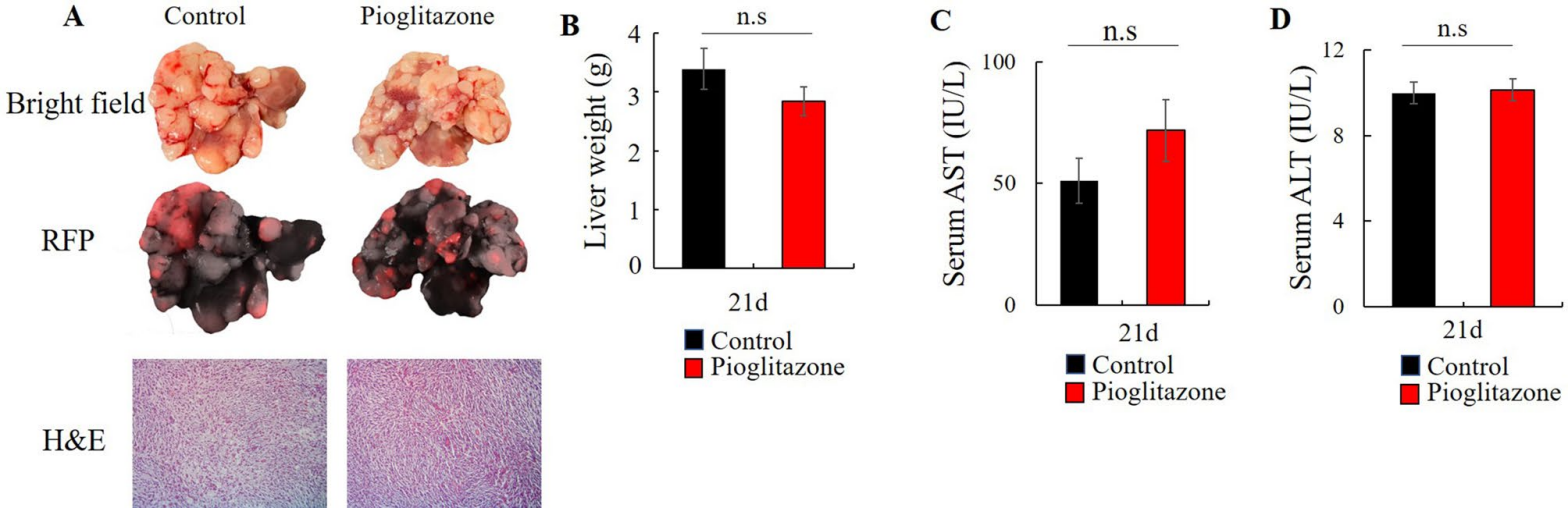
Effect of pioglitazone on hepatic metastasis after IRI. (**A**) Macroscopic and microscopic evaluation in the control and pioglitazone groups 21 days after spleen injection of cancer cells and IRI. Liver sections from treated and untreated mice were stained with hematoxylin–eosin (H&E) (after 21 d) (**B**) mouse liver weight at day 21 (n = 7/group). Data represent mean ± SD. Serum level of AST and ALT were measured with standard kits at 21 d (*n* = 7) (**C** and **D**). Values represent the mean ± SD.

We determined the effect of Pioglitazone on IRI-induced liver metastasis compared to vehicle control. Liver colon-cancer metastasis was resistant to pioglitazone (Fig. 5A,B). Liver metastasis was detected by RFP fluorescence in the liver (Fig. 5A). The increased liver weight of the mice was due to metastasis (Fig. 5A,B). Pioglitazone also had no effect on tumor histology (Figure 5A), and no effect on AST and ALT at day 21 (Fig. 5C,D).

## Discussion

In the present study, we showed that IRI raised the serum levels of AST (Fig. 2A), ALT (Fig. 2B), MMP-9 (3A), pro-MMP-9 (Fig. 3B), IL-6 (Fig. 4A) and TNF-α (Fig. 4B) within 24 h.

Previous reports demonstrated that pioglitazone inhibited IRI-induced MMP-9 elevation^5,8^. Some previous reports suggested PPARγ agonists inhibited colon cancer growth^9–11^ and liver metastasis^12^. On the other hand, some reports suggested that PPARγ agonists promotes tumor growth^17–19^. In the present study pioglitazone had no effect of the very extensive metastasis observed. In the IRI model, previous reports stated that the attenuation of the inflammatory response or metastasis by PPARγ was only for a short time^8,16^. In the present study, serum AST levels significant decreased and ALT tended to decrease by 24 h after pioglitazone treatment of IRI mice compared with control mice. (Fig. 2A). Pioglitazone had no effect of the IRI-induced increases in IL-6, TNF-α and MMP-9 (Fig. 3AB, Fig. 4B, C), and no effect on AST and ALT on day 21 (Fig. 5C,D).

In our previous study, pioglitazone overcome doxorubicin resistance in a patient-derived orthotopic xenograft (PDOX) model by down-regulating p-glycoprotein expression^18^ and had similar effects against another cisplatin- resistant PDOX model^19^. Some effects of pioglitazone are not relevant in the present studies. Even though pioglitazone could inhibit liver injury in the present studies, it could not inhibit factors associated with inflammation such as IL-6 or TNF-α and did not inhibit the very expensive liver metastasis observed. In the present study, tumor growth was effected by splenic injection of cancer cells which was shown to induce high levels of liver metastasis due to the association of cancer cells with splenocytes^20–23^. Our hypothesis is that pioglitazone may have different effects depending on route of administration and schedule. Future studies will further investigate at the relationship of IRI and metastasis and its inhibition and measure liver enzymes over longer periods.
